# Genetic Insight into Expression-Defined Melanoma Subtypes and Network Mechanisms: An in Silico Study

**DOI:** 10.3390/genes16121428

**Published:** 2025-11-30

**Authors:** Desirèe Speranza, Mariapia Marafioti, Martina Musarra, Vincenzo Cianci, Cristina Mondello, Maria Francesca Astorino, Mariacarmela Santarpia, Natasha Irrera, Mario Vaccaro, Nicola Silvestris, Concetta Crisafulli, Marco Calabrò, Silvana Briuglia

**Affiliations:** 1 Department of Chemical, Biological, Pharmaceutical and Environmental Sciences, University of Messina, 98125 Messina, Italy; desiree.speranza@gmail.com; 2 Department of Clinical and Experimental Medicine, University of Messina, 98125 Messina, Italy; natasha.irrera@unime.it (N.I.); mario.vaccaro@unime.it (M.V.); 3 School of Specialization in Medical Oncology, Department of Human Pathology “G. Barresi”, University of Messina, 98125 Messina, Italy; marafiotimariapia@gmail.com (M.M.); martimusa.mm@gmail.com (M.M.); 4 Medical Oncology Unit, Department of Human Pathology “G. Barresi”, University of Messina, 98125 Messina, Italy; mariacarmela.santarpia@unime.it; 5 Department of Biomedical and Dental Sciences and Morphofunctional Imaging, University of Messina, 98125 Messina, Italy; maria.astorino@studenti.unime.it (M.F.A.); concetta.crisafulli@unime.it (C.C.); marco.calabro@unime.it (M.C.); silvana.briuglia@unime.it (S.B.); 6 Medical Oncology Department, IRCCS Istituto Tumori “Giovanni Paolo II”, 70124 Bari, Italy; n.silvestris@oncologico.bari.it

**Keywords:** melanoma, genetic heterogeneity, expression-based stratification, lipid metabolism

## Abstract

Background: Melanoma is a highly heterogeneous neoplasia in which transcriptional profile encodes much of the biological diversity that determines tumor progression and therapeutic response. To refine its molecular stratification and profiles characterization, we conducted an in silico transcriptomic analysis. Methods: Public microarray datasets from the GEO and ArrayExpress were examined, and the E-MTAB-6697 expression dataset was selected. We used a K-Means clustering algorithm to stratify 194 tumor samples into expression-driven subgroups and analyzed each one to define their transcriptional and biological profiles. Differential expression analysis between identified clusters and controls was performed. Additionally, we applied Weighted-Gene correlation analysis to identify coordinated expression hubs in the tumor dataset and tested the resulting modules for correlation with the identified clusters. Results: Unsupervised clustering of melanoma transcriptomic profiles identified three distinct molecular subtypes characterized by divergent biological programs. While all clusters shared the dysregulation of pathways involved in epidermal differentiation, immune response, and lipid metabolism, they diverged in proliferation, phenotypic plasticity, metabolic adaptation, and apoptotic regulation. Cluster A was characterized by enrichment in DNA replication, repair, and mitochondrial metabolism modules, suggesting a proliferative yet genomically stable state. Cluster B showed enrichment in immune and cytokine signaling pathways alongside reduced proliferative activity, consistent with a quiescent or transitional phenotype. Cluster C displayed coordinated enrichment in cell-cycle, DNA-maintenance, and neuroectodermal reprogramming pathways, indicating a highly plastic and proliferative subtype. Despite these molecular distinctions, all clusters retained an “immunologically hot” profile (IPS 7–8), indicating potential responsiveness to immunotherapy. Conclusions: These findings provide an overview of the functional characteristics of melanoma heterogeneity and identify biological processes that could be targeted by drugs for the development of tailored therapies for each subtype. Nevertheless, future studies in independent clinically annotated cohorts would be required.

## 1. Introduction

Cutaneous melanoma (CM) represents a significant public health burden, currently ranking as the fifth most frequently diagnosed malignancy in the United States, with approximately 100,000 new cases reported annually [1], and it is the sixth most common cancer in developed countries [2].

Global incidence rates vary, with the highest reported in Australia at 37 per 100,000, whereas Europe and the United States report lower rates of approximately 25 and 30 per 100,000, respectively [3].

Accurate disease staging is crucial for proper diagnosis, prognosis, and treatment selection. The TNM system evaluates both clinical and pathological features [4], including tumor thickness (measured by Breslow index), ulceration, lymph node involvement, and distant metastasis. Greater tumor thickness and ulceration are associated with a worse prognosis and increased melanoma spread [5]. Lymph node involvement indicates local spread, while metastasis refers to distant dissemination, commonly affecting the lungs, liver, brain, bones, and skin [4].

Due to the heterogeneous nature and multifactorial etiology of this disease, the risk of melanoma is influenced by a complex and dynamic interaction between genetic, phenotypic, and environmental factors [6].

In this context, The Cancer Genome Atlas (TCGA) project classifies cutaneous melanoma into four major genomic subtypes based on driver mutations: *BRAF*-mutant, *RAS*-mutant, *NF1*-mutant, and triple wild-type [7]. Among these, *BRAF* mutations—primarily V600E, but also V600K, V600R, and K601E—are the most common and mutually exclusive with *RAS* hotspot mutations. In contrast, non-hotspot *BRAF* mutations can co-occur with mutations in *NRAS, KRAS, HRAS,* or *NF1*, all of which converge on the MAPK (RAS–RAF–MEK–ERK) pathway and often also activate PI3K/AKT signaling [8].

*NF1* mutations, seen in ~15% of melanomas—mostly in older individuals with high mutational burden—frequently result in loss of function, promoting MAPK hyperactivation. The triple wild-type subtype, which lacks *BRAF*, *NRAS*, and *NF1* mutations, often features alterations in *GNAQ*, *GNA11*, *KIT*, *CTNNB1*, or *EZH2*, and is typically driven by structural genomic aberrations, including copy number variations and gene fusions rather than UV-induced point mutations.

Importantly, three out of four subtypes converge on MAPK pathway hyperactivation, establishing it as a central driver in melanoma development and progression [7]. In parallel, *TERT* promoter mutations, found across all subtypes, enhance telomerase activity and contribute to replicative immortality [9].

From a clinical perspective, the *BRAF*-mutant subtype is often more responsive to targeted therapies (e.g., BRAF and MEK inhibitors), showing improved outcomes. However, resistance frequently emerges via reactivation of the MAPK pathway [8]. Conversely, *NRAS*-mutant and *NF1*-mutant melanomas are more aggressive and linked to poorer prognosis, while triple wild-type cases generally respond less favorably to immunotherapy [10].

Beyond MAPK, dysregulation of the PI3K/AKT/mTOR axis—often through mutations or amplifications—also supports tumor growth and therapeutic resistance, making it a potential target for future treatments [11].

At the phenotypic level, melanoma exhibits high plasticity through epithelial-to-mesenchymal transition (EMT)-like programs, despite its non-epithelial origin. This is driven by dynamic shifts between a proliferative (*MITF*-high) and an invasive (*AXL*-high) state—a process known as phenotype switching [12]. Key EMT regulators (e.g., SNAIL, TWIST, ZEB), often influenced by the tumor microenvironment, modulate transcription and epigenetic states that enhance invasiveness, immune evasion, and resistance to both targeted therapies and immunotherapy [13].

Immune evasion, recognized as a hallmark of cancer, refers to the ability of tumor cells to evade immune recognition and destruction through a complex interplay of molecular alterations, cellular adaptations, and modulation of the tumor microenvironment. Although melanoma is considered highly immunogenic due to its high mutational burden, which leads to the generation of numerous neo-antigens capable of triggering an immune response, melanoma cells often manage to evade immune surveillance through several sophisticated mechanisms. Tumors achieve this by upregulating immune checkpoint molecules such as PD-L1, CTLA-4, LAG3, TIM3, and TIGIT, leading to T-cell exhaustion and suppression of immune surveillance [14]. Other mechanisms include β2-microglobulin (B2M) loss, MHC-I downregulation [15], IDO1-mediated tryptophan catabolism, and secretion of immunosuppressive cytokines like TGF-β1 and IL-10 [16]. Moreover, activation of STAT3, a key oncogenic transcription factor, further supports EMT, metastasis, and immune suppression [17].

Beyond somatic mutations, several germline variants have been associated with an increased risk of developing CM, in particular for hereditary cases. These genes are involved in various biological processes such as cell cycle regulation and telomere maintenance, and are categorized by penetrance into high, medium, and low.

Melanoma susceptibility genes are commonly classified according to their penetrance. High-penetrance genes such as *CDKN2A*, *CDK4*, *BAP1*, *POT1*, and *TERT* are defined by a relative risk (RR) exceeding 5, and account for a substantial fraction of familial cases [18,19].

Medium- and low-penetrance alleles are instead prevalent in the general population and, separately, rarely confer sufficient risk to cancer development. Medium-penetrance genes, with RR estimates ranging from 2 to 5, confer an intermediate increase in melanoma risk, particularly in the presence of synergistic effects among multiple susceptibility alleles. Low-penetrance genes, characterized by RR values below 2, contribute to the polygenic background of melanoma susceptibility [6].

The potential cumulative effect of multiple alleles, and their interaction with environmental exposures, can synergistically increase melanoma susceptibility beyond a pathogenic threshold and are also a relevant source of complexity in understanding the biological background behind the disease [18].

Considering the high variability and the large number of high-, medium-, and low-risk genes that may be involved in CM, it does not come as a surprise that melanomas, despite sharing some characteristics, are highly heterogeneous [20,21,22,23].

Indeed, despite shared driver mutations and histopathological features, melanomas represent a highly heterogeneous group of tumors, differing in clinical course, response to therapy, and underlying molecular programs.

Interestingly, it has been observed that the heterogeneity of CMs can be largely explained by transcriptional differences and regulation, which drive distinct cell states, phenotypic plasticity, and therapy responses [24,25,26,27].

Transcriptional differences may be a central driver of melanoma heterogeneity; understanding these differences is crucial not only to better understand the pathology, etiology, and progression, but also for developing more effective, personalized treatment strategies. Nevertheless, the precise events and biological processes implicated are yet to be fully characterized. For these reasons, in this exploratory study we tried to increase our understanding of CM heterogeneity and its relationship to genetics. In particular, we focused on expression variability as a possible marker to define different subtypes among CM cases. To explore this hypothesis, we selected a relatively large sample of CM data from publicly available repositories and tried to identify potential sub-clusters within this population and pinpoint the genetic expression differences discriminating against such sub-types.

## 2. Materials and Methods

**Figure 1:**
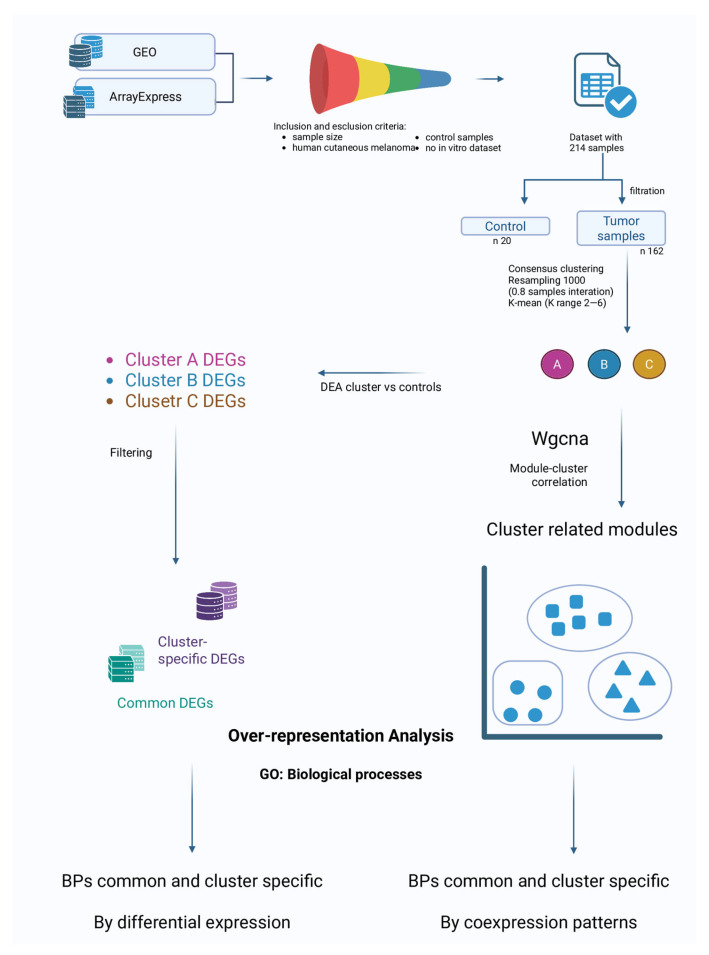
Flowchart of study.

In Figure 1 we present the flowchart of study.

### 2.1. Dataset Search and Selection

Systematic research was performed across the publicly accessible databases Gene Expression Omnibus (GEO) [28] and ArrayExpress [29]. Inclusion criteria were (i) adequate sample size, (ii) availability of data from human cutaneous melanoma (excluding in vitro studies), and (iii) presence of a proper control cohort. The search aimed to minimize potential variability due to factors not related to gene expression. All the databases were accessed on 4 August 2025 to identify appropriate datasets. Although multiple datasets were initially evaluated, we selected one that most appropriately satisfied these inclusion criteria, and it exclusively comprised samples of cutaneous melanoma. Therefore, the present analysis is specifically representative of cutaneous melanoma.

### 2.2. Probes and Genes Annotation

The probe-to-gene conversion was manually curated using the GPL570 platform annotation data (https://www.ncbi.nlm.nih.gov/geo/query/acc.cgi?acc=GPL750 (accessed on 8 August 2025)), which corresponds to the array reported by the original authors (Affymetrix Human Genome U133 Plus 2.0 Array). From the GPL750 annotation, control probes were identified and removed from the expression matrix. Then, we generated a probe-to-gene (Ensembl ID) conversion table, through which we assigned an Ensembl ID to each probe. When multiple probes were mapped to the same gene, probes were collapsed, and the one with the maximum average expression value across samples was selected as the representative. This approach was preferred over the mean as it avoids dilution from poorly performing probes and is usually considered the most reliable choice [30]. Any data displaying inconsistencies or deemed unreliable was eliminated in this step. Finally, Ensembl IDs were converted to Gene Symbols. The processed expression matrix was used for further analyses. These steps were performed using a Python v3.13.5-based pipeline, in particular the Ensembl ID to Gene Symbol conversion was carried out with the mygene v.3.2.2 package [31].

### 2.3. Clustering of Melanoma Samples

To evidence possible melanoma subtypes in the analyzed dataset we opted to perform a clustering analysis on the pathologic samples of the database. The first step was to remove the control samples (20 samples) data from the expression matrix, to obtain a tumor-positive-only sub-sample (194 samples). Additionally, any non-melanoma sample was filtered out in this step. To reduce computational load before the clustering step, the high-dimensional gene expression matrix was reduced using UMAP (Uniform Manifold Approximation and Projection), which preserves both local and global data structure in a low-dimensional space. UMAP was chosen after testing PCA (Principal Component Analysis) and t-SNE (t-distributed Stochastic Neighbor Embedding), as it provided the clearest separation of sample groups in low-dimensional visualizations. We applied the consensus clustering framework [32] to perform clustering. Consensus clustering is a resampling-based method that repeatedly applies a clustering algorithm to random subsets of the data, producing a consensus matrix that quantifies how often each pair of samples clusters together across resamples. This approach reduces sensitivity to random initialization and sampling noise and provides a robust estimate of cluster stability [32]. K-Means clustering was used as the base algorithm within consensus clustering. K-Means partitions samples into k clusters by iteratively assigning each sample to the nearest cluster centroid and updating centroids as the mean of the assigned samples, minimizing within-cluster variance. The number of k clusters was tested in the range 2–6, and the optimal cluster number was determined by the maximum increase in the consensus CDF (Cumulative Distribution Function) area. To further evaluate stability, we computed the Proportion of Ambiguous Clustering (PAC), defined as the fraction of consensus values in the interval (0.1–0.9), where values near 0 or 1 represent stable sample assignments and intermediate values indicate uncertainty. Lower PAC scores reflect greater stability. Finally, silhouette scores were calculated on the reduced UMAP space to quantify cluster compactness and separation, with higher values indicating better-defined clusters. Consensus clustering was performed with 1000 resamplings per k, generating consensus matrices that quantify how consistently pairs of samples cluster together across iterations.

The final cluster assignments were obtained by applying K-Means on the reduced UMAP dimensions (150 components) with the selected best cluster number. The clustering step was performed in the Python v.3.13.5 environment, using the following packages: umap-learn v.0.5.9.post2 [33], scikit-learn v.1.7.1 [34], pandas v.2.3.2 [35], and numpy v.2.2.6 [36]. The resulting clusters were used for downstream analyses.

### 2.4. Clusters vs. Control: Differential Expression and Over-Representation Analysis

After assigning tumor samples to consensus clusters, the previously removed normal samples were reintroduced into the dataset. Differential Expression Analysis (DEA) was then performed for each cluster against the control samples using the BioTEA v.2.0.0 package [37]. Rank Product (RankProd package v.3.22 in R environment) was applied for DEA [38]. We opted to use RankProd as it is better suited for cross-dataset comparisons as it evaluates gene ranks rather than absolute expression values. Since the dataset we selected is a collection of data from multiple databases, we deemed RankProd as the most optimal choice. In our analysis we accepted as Differentially Expressed Genes (DEGs) all the genes with an adjusted *p*-value < 0.05. The adjusted *p*-value was calculated by correcting the *p*-value with the False Discovery Rate (FDR) method to reduce the number of false positives. The lists of DEGs generated for each comparison were then used to highlight genes specifically dysregulated in one cluster and DEGs common to all clusters. Cluster-specific and common DEGs lists were used as input for Gene Ontology (GO) enrichment analysis to identify statistically significant alterations in gene ontologies. The GSEApy v.1.1.9 package [39] was utilized for enrichment analysis.

### 2.5. In-Between Clusters Characteristics: Weighted-Gene Co-Expression Network Analysis

To further explore the biological differences among the identified melanoma clusters, we performed Weighted-Gene Co-expression Network Analysis (WGCNA). WGCNA is a systems biology approach that constructs gene co-expression networks based on pairwise correlations, grouping genes into modules of highly co-expressed genes [40]. Unlike DEG analysis, which identifies genes that are differentially expressed between groups, WGCNA is used to highlight genes whose expression is coordinated. The analysis was carried out on the full expression matrix of pathological samples to define modules of co-expressed genes. Each module was then correlated with the clusters identified from the clustering step, with the aim of finding gene networks associated with specific subtypes. Finally, for modules showing the strongest correlations, we performed functional enrichment analysis to identify GO Biological Processes and pathways underlying these transcriptional programs. The package pyWGCNA v.2.2.1 [41] was used to perform this analysis.

All the analyses and the images produced were performed in a Python environment. For visualization matplotlib v.3.10.5 [42] and seaborn v.0.13.2 [43] packages were used.

## 3. Results

In the present study, we analyzed an expression dataset containing information derived from pathological tissues and normal tissues from patients with a diagnosis of melanoma and controls. The main aim of study was to find potential subtypes of the pathology based on genetic criteria, and their characterization.

### 3.1. Dataset Selection

After the search performed on GEO [28] and ArrayExpress [29] repositories, we ultimately selected the dataset ID: E-MTAB-6697 [https://www.ebi.ac.uk/biostudies/arrayexpress/studies/E-MTAB-6697 (accessed on 8 August 2025)] from ArrayExpress. E-MTAB-6697 is a collection of data from a total of 214 samples which include both primary tumors and tumor-free melanoma tissues. The rationale behind the selection of this dataset is that it comprises the data of four independent studies (GSE19234, GSE23376, GSE7553, and GSE15605), providing a sample size sufficiently robust to bolster the statistical robustness of our analyses. Further, the data in the collection was normalized and batch-corrected, making it ready for further analyses without needing extensive preprocessing. Additionally, the datasets in the collection were all generated from the GPL570 platform (Affymetrix Human Genome U133 Plus 2.0 Array), thus minimizing potential variability due to different platforms. Further details on the dataset are publicly available on the ArrayExpress database.

### 3.2. Clustering

Before the clustering step, the expression matrix was preprocessed to remove data from normal samples. This step reduced the number of samples to 194. Additionally, non-melanoma samples (32) were also filtered out. The final sample size was 162 melanoma-related samples. The expression matrix dimensionality was then reduced through UMAP, and the resulting matrix was used as input for clustering based on the consensus clustering network and using the K-Means algorithm (please refer to Section 2.3).

According to the stability metrics used, we retained k = 3 as the most robust and interpretable subtype definition.

The clustering generated three subsamples containing 51, 52, and 59 samples, respectively.

### 3.3. Differential Expression Analysis (DEA)

Analyzing DEGs between cluster-specific samples and controls enables the identification of genes involved in the subtype characterization. The DEA was performed on each cluster vs. controls independently.

**Figure 2:**
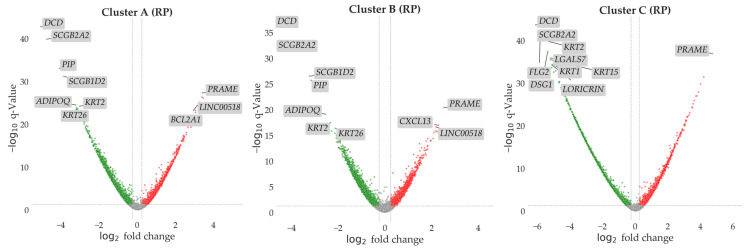
In the volcano plot we report all the genes explored in the DEA for each cluster vs. control comparison performed with the Rank Product (RP) approach. Sample sizes were 51 for Cluster A, 52 for Cluster B, 59 for Cluster C, and 20 for controls. On the *y*-axis we report the log_10_ value of the adjusted *p*-value. The dashed line shows the significance threshold of *p* = 0.05. All the genes above this line are considered differentially expressed. On the *x*-axis we report the log_2_ fold change. The dashed lines show the fold change thresholds (fold change > |0.5|) used in the analysis. Up- and downregulated DEGs are reported in red and green, respectively. Genes not significantly dysregulated are reported in grey. Top 10 DEGs by significance are labeled in the plots for each comparison.

The DEA considered as upregulated all the DEGs with a statically supported increased expression in the cluster groups compared to the controls for each cluster. The downregulated DEGs are intended as the gene whose expression was inferior in the clusters groups compared to controls for each cluster. The statistical significance was corrected using FDR method to reduce the false positive rate. DEGs with an FDR-adjusted *p*-value < 0.05 were considered statistically significant. Only genes whose fold change was greater than 0.5 (absolute value) were selected. Volcano plots summarizing the results from DEA for each comparison are reported in Figure 2.

### 3.4. DEGs Comparison

Data obtained from DEA analyses was processed and filtered to select genes that were identified as cluster-specific DEGs, plus the genes that were differentially expressed in all clusters. The numbers of DEGs up- and downregulated from DEA are reported in Table 1.

**Figure 3:**
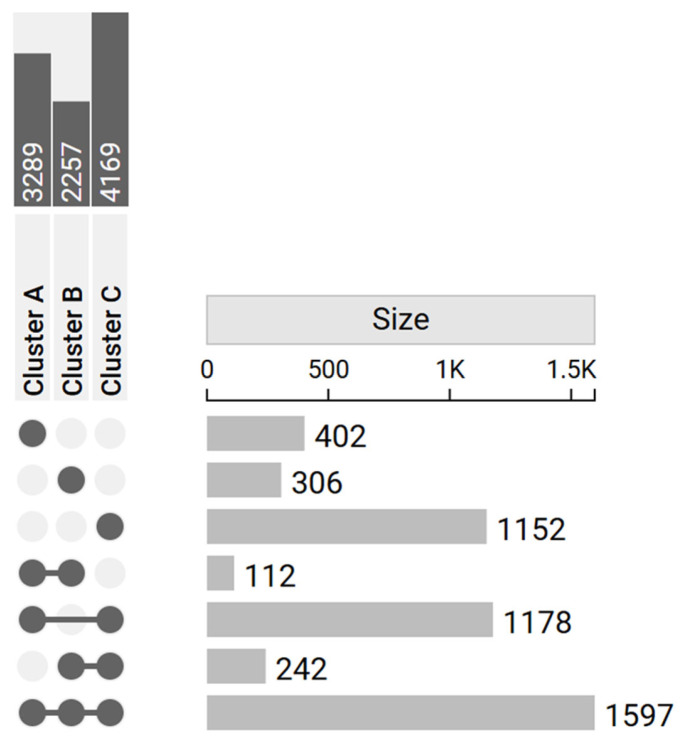
Upset plot reports the number of DEGs in each cluster (dark gray in the upper left corner) and their intersections (light gray in the right-hand side of the plot). The matrix layout on the left displays the combinations of sets involved in each intersection: filled dots connected by lines represent the specific clusters’ intersections, while unconnected or empty dots indicate absence from a given cluster. On the right, bars quantify the number of elements belonging to each intersection. Specific DEGs were 402 for Cluster A, 306 for Cluster B, and 1152 for Cluster C.

From the initial results, 402 genes were identified as DEGs only in Cluster A, 306 only in Cluster B, and 1152 only in Cluster C. Additionally, a total of 1597 genes were identified as DEGs in all the clusters. The upset plot (Figure 3) summarizes the number of DEGs across clusters and their intersections.

### 3.5. Over-Representation Analysis (ORA)

**Figure 4:**
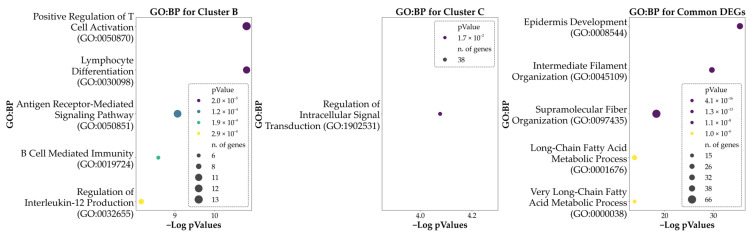
Bubble plots of the top five significant Gene Ontology: Biological Processes (GO: BP) associated with each cluster and with the genes found to be DEGs in all the clusters. Cluster A is not represented here as no significant GO: BP resulted from the ORA. Hue reports the *p*-values of the analysis, while the size of each bubble indicates the number of DEGs being part of each process.

The identified cluster-specific and common DEGs were then used as input for ORA to highlight the predominant GO characterizing the clusters. The analysis was based on the biological process (BP) entries to provide a biological perspective on the functions associated with each subtype identified (cluster-specific DEGs) and to shed light on the common genetic background at the basis of the phenotype (by evaluating the common DEGs). The ORA conducted using the 402 DEGs associated with Cluster A revealed no significant enrichments for any BP. The ORA conducted using the 306 DEGs associated with Cluster B revealed a significant enrichment for 66 BPs. The ORA conducted using the 1152 DEGs associated with Cluster C revealed a significant enrichment for 1 BP. Finally, the ORA on the 1597 DEGs shared by the three clusters revealed a significant enrichment in 218 BPs. In all cases, the enriched ontologies were determined based on the significance of FDR corrected *p*-values, highlighting those with the most statistically significant associations. The analysis provided information on the DEGs and the related biological pathways characterizing the unique features of the clusters and the underlying common elements. In Figure 4 we report the top five enriched ontologies obtained from the results of each ORA. Table 2 reports a summary of the results of the aforementioned ontologies, while Supplementary Table S2 provides a comprehensive list of all enriched GO terms, along with their associated DEGs.

### 3.6. Weighted-Gene Co-Expression Network Analysis (WGCNA) and Module Correlation with the Clusters

As a further step to characterize the identified clusters, we proceeded to apply WGCNA to the expression matrix of the pathological sample to identify modules of genes co-expressed in the sample (please refer to Section 2.5). Then, the modules were tested for correlation with the clusters to highlight the ones better characterizing the modules differences. From the analysis we identified a total of 15 different modules. In Supplementary Figure S1 we summarize the clustering of the module eigengenes.

**Figure 5:**
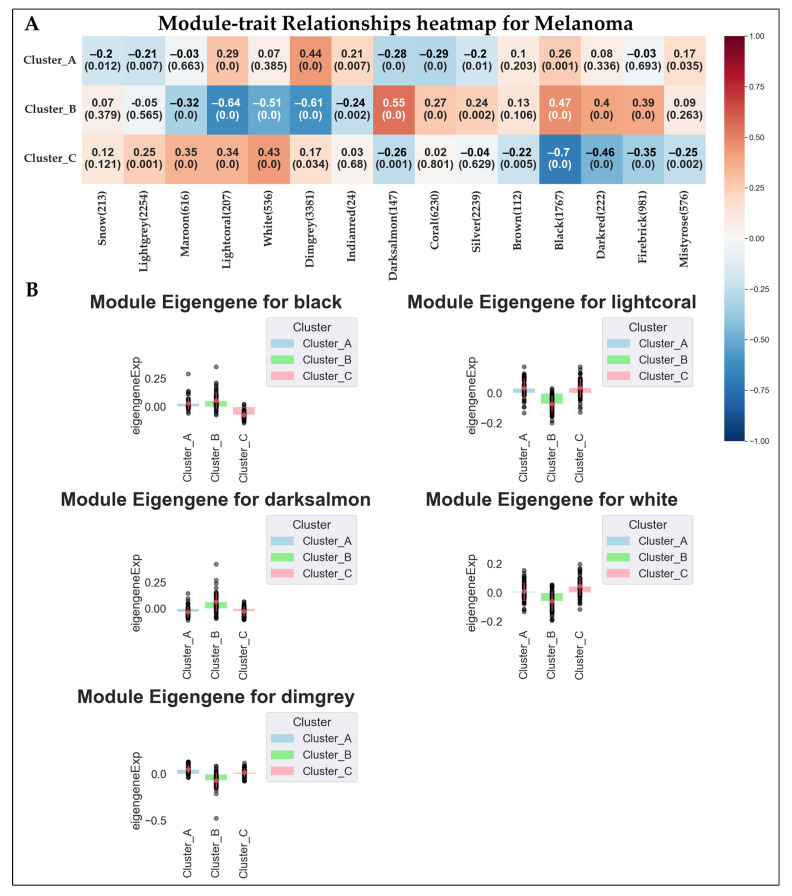
Here we report (**A**) the results of the module–cluster relationships. For each correlation we show the correlation coefficient and the *p*-value of the analysis. (**B**) Bar plot showing the expression behavior of the top five modules significantly associated with the clusters. The black dots represent the module eigengene values of individual samples within each cluster.

The modules were then tested for correlation with the clusters previously identified. A cluster-associated module may suggest a co-expressed gene network that is systematically linked to the subtype, providing further information about its underlying biological mechanism. Among the modules, the Lightcoral, White, Dimgrey, Darksalmon, and Black modules showed a significant correlation with at least one of the clusters, using a restrictive *p*-value cut-off of *p*-value < 0.001 and a r value > 0.5. Of these modules, Lightcoral (Cluster A r = 0.29, Cluster B r = −0.64, Cluster C r = 0.34) and White (Cluster A r = 0.07, Cluster B r = −0.51, Cluster C r = 0.43) showed the highest positive correlation with Cluster C and the highest negative correlation with Cluster B; Dimgrey (Cluster A r = 0.44, Cluster B r = −0.61, Cluster C r = 0.17) showed the highest positive correlation with Cluster A and the highest negative correlation with Cluster B; Darksalmon (Cluster A r = −0.28, Cluster B r = 0.55, Cluster C r = −0.26) showed the highest positive correlation with Cluster B and a low-moderate negative correlation with Cluster A and C; and Black (Cluster A r = 0.26, Cluster B r = 0.47, Cluster C r = −0.70) showed the highest positive correlation with Cluster B and the highest negative correlation with Cluster C. Figure 5 reports the modules–clusters relationships.

Similarly to DEA analyses, we used data from top modules to highlight the predominant GO characterizing the clusters. In particular, we found 31 BPs enriched in the Black Module, 348 BPs in the Dimgrey module, 169 BPs in the Lightcoral module, and 191 in the White module. No significant BPs were found for the Darksalmon module.

**Figure 6:**
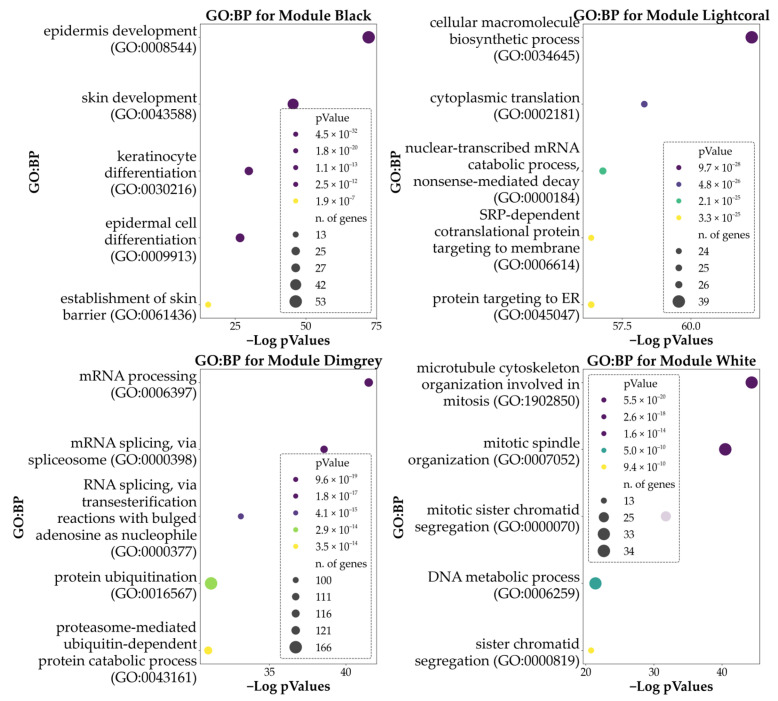
Here are reported the bubble plots of the top five significant GO: BPs associated with the genes of the top five modules derived from WGCNA. Hue reports the *p*-values of the analysis, while the size of each bubble indicates the number of DEGs being part of each process. Please note that enrichment for the module Darksalmon retrieved no significant BPs.

Data obtained is summarized in Figure 6, which shows the top five (by significance) GO: BPs associated with the top four modules. Supplementary Table S2 reports the full details of the enrichment analysis for the top four modules.

## 4. Discussion

Melanoma remains a severe issue in modern society. It has a highly heterogeneous nature, influenced by numerous factors, including genetic, phenotypic, and environmental factors. And this complexity severely impacts its progression and impairs potential treatments. A more detailed characterization of this disease could provide new avenues for more effective and personalized treatment strategies. With this aim, in this paper we tackled the issue of heterogeneity, trying to identify possible melanoma subtypes based on gene expression data, since several literature sources have shown that expression may explain a large part of melanoma heterogeneity [24,25,26,27].

From our analyses we highlighted biological processes that are altered in melanoma in general, and some others whose alteration may be typical of specific melanoma subtypes.

Overall, the biological processes we found in our analysis can be categorized into a few main groups that influence (1) differentiation, (2) proliferation, (3) phenotypic switching and invasiveness, (4) inflammation and immune responses, (5) general alteration of basic cellular functions such as replication, transcription, and translation, and (6) lipid metabolism. From a modular perspective, the white module represents a central proliferative-DNA-maintenance hub, while dimgrey captures transcriptional and RNA-processing machinery supporting active biosynthesis. The lightcoral module integrates metabolic and proteostatic stress responses, including ER–Golgi transport, autophagy, and oxidative stress management. Conversely, the black module reflects epithelial differentiation and lipid-metabolic processes, inversely correlated with proliferative and inflammatory programs.

In the following section we will discuss in more depth our results and their potential implications with melanoma.

### 4.1. Biological Processes Involved in Differentiation

Processes such as Epidermis Development (GO:0008544), Epithelium Development (GO:0060429), Epithelial Cell Differentiation (GO:0030855), Extracellular Matrix Organization (GO:0030198), and Skin Epidermis Development (GO:0098773), which all point to processes involved in differentiation and proliferation, were found to be significantly dysregulated in all subtypes found in our analysis compared to normal tissue.

The enrichment of these categories reflects the dysregulation of transcriptional programs typical of keratinocytes (e.g., SPRR1/2/3, KRT10/KRT16, LOR, IVL, EVPL, SCEL, TGM1/TGM3, CERS3, ACER1, DSP) and, above all, the epithelial crosstalk that influences melanoma biology. The loss of control exerted by keratinocytes over melanocytes is a well-documented element of melanoma genesis and progression [44]. Indeed, markers of keratinocyte differentiation and epidermal development are associated with disease stage and prognosis. Elevated levels of KRT1/5/6/14/15/16/17 have been found to correlate with lower overall survival in melanoma patients, confirming that the epidermis/keratinocyte signature can also provide information on outcome [45].

Furthermore, in vivo and ex vivo models have demonstrated that differentiated keratinocytes actively promote melanoma progression [46]. Unlike undifferentiated cells, differentiated keratinocytes induce melanoma invasion through a Notch-dependent pathway, involving inhibition of *MITF*, a gene found to be dysregulated across all analyzed melanoma subtypes. This confirms their role as dynamic regulators of epidermal homeostasis. Consistently, melanocytes in contact with undifferentiated keratinocytes maintain a normal phenotype, whereas interaction with differentiated keratinocytes drives them toward nevus-like or melanoma-like characteristics [46].

This finding underscores how epidermal differentiation and developmental processes can intersect with the aggressive phenotypic trajectories of melanoma [46]. In this context, the functional nodes present in our lists, such as WNT5A, EPHA2, and EGFR/ligands (EREG), link epidermal signaling to invasion and therapeutic tolerance. WNT5A promotes motility and invasion, as well as contributing to resistance to BRAF inhibitors [47,48], while EPHA2 mediates resistance to vemurafenib and a therapy-induced metastatic phenotype [49,50]. Furthermore, epigenetic activation of EGFR has been associated with resistance to BRAF inhibitors, and in some studies, increased EGFR copy number or expression has been linked to poor prognosis [51,52].

Moreover, UV-induced epigenetic alterations in keratinocytes and melanocytes affect pigmentation and remodel the epidermal microenvironment, potentially promoting tumor development. The epidermis, enriched in adhesion molecules such as cadherins, integrins, immunoglobulin family members, and selectins, maintains tissue homeostasis through adhesion-dependent and -independent mechanisms. Dysregulation of these molecules can alter melanoma cell migration, immune cell access, and intracellular signaling, influencing proliferation, survival, and immune evasion. Clonal expansion of UV-mutated keratinocytes may co-evolve with melanocytes in pro-melanoma niches, and recent evidence suggests that mutations in desmosomal components may be linked to melanoma progression [53].

The WGCNA revealed that epidermal and differentiation-related pathways are associated with the “Black” module, which is upregulated in Cluster B and downregulated in Cluster C. Although these processes appeared dysregulated across all melanoma subtypes, their specific modulation within the “Black” module highlights subtype-dependent differences. The upregulation of this module in Cluster B suggests a more aggressive phenotype, characterized by enhanced activation of epidermal pro-tumoral signaling and stronger epithelial–melanocyte interactions that may promote invasion, metastasis, and therapeutic resistance. Conversely, the downregulation observed in Cluster C indicates a subtype with reduced involvement of these epidermal pathways, potentially corresponding to less aggressive biological behavior.

From a therapeutic perspective, targeting the molecular components of the “Black” module, such as WNT5A, EPHA2, and EGFR, could offer promising strategies to counteract tumor invasiveness and overcome drug resistance, particularly in Cluster B patients, who may be more responsive to such inhibitors. Data from the literature already highlights the potential of WNT5A downregulation to reduce melanoma’s invasive properties [54,55,56]. Additionally, inhibitors of EGFR or EPHA2, or agents that modulate WNT5A signaling, may therefore represent potential adjuvant approaches to improve therapeutic efficacy and patient outcomes in the melanoma subtypes with heightened epidermal signaling activity, as in the identified Cluster B. Nevertheless, more targeted studies would be needed to confirm such observations.

### 4.2. Proliferative and Pro-Survival Biological Processes

Regarding proliferation, multiple pathways related to proliferation were found to be dysregulated, such as microtubule cytoskeleton organization involved in mitosis (GO:1902850), mitotic spindle organization (GO:0007052), DNA metabolic process (GO:0006259), mitochondrial translation (GO:0032543), mitochondrial translational elongation (GO:0070125), mitochondrial translational termination (GO:0070126), DNA metabolic process (GO:0006259), and DNA replication (GO:0006260). Interestingly, these processes did not result from the comparison with normal tissues, but rather they derive from a transcriptional module (Dimgrey), which is differently expressed in the diverse subtypes. Indeed, the Dimgrey module is upregulated in Cluster A and downregulated in Cluster B, suggesting that of the three clusters, Cluster A may include tumoral samples with heightened proliferative properties and enhanced tolerance to DNA damage. It should be noted that the DNA metabolic process (GO:0006259) and DNA replication (GO: 0006260) are enriched in the White module; while GO:0006259 is correlated with the Lightcoral module. Consistently with the previous observation, both modules are downregulated in Cluster B and have a slight upregulation in both Clusters A and C. From a clinical perspective, Cluster A is characterized by tumors with high proliferative activity and increased tolerance to DNA damage, indicating a more aggressive phenotype and reduced sensitivity to conventional chemo- and radiotherapy. In contrast, Cluster B shows reduced proliferation and downregulation of related transcriptional modules, suggesting a more quiescent phenotype that may respond more effectively to differentiation-based therapeutic strategies. The concurrent activation of DNA repair mechanisms and mitochondrial metabolic pathways in these tumors may represent an important mechanism of therapeutic resistance.

From a therapeutic standpoint, targeting DNA repair pathways could be particularly beneficial. Likewise, the inhibition of mitochondrial metabolism (i.e., through OXPHOS inhibitors) may reduce both the proliferative potential and survival of melanoma cells with elevated mitochondrial activity [57,58]. Finally, rational combination therapies integrating replication and metabolic inhibitors with immunotherapy may enhance clinical efficacy, especially in highly proliferative subtypes such as Cluster A.

Overexpression of genes involved in the above biological processes, including *ORC1/5, CDC6/7, MCM4, PCNA, RFC3/4, RPA1–3, PRIM1/2, POLA2, POLE2, PRIMPOL, POLH, TIMELESS/TIPIN*, and *WDHD1*, is related to an increase in replication stress tolerance through replication fork protection and genomic stability [59,60,61,62]. Concurrent activation of homologous recombination (RAD51, RAD51B/C, RAD54L, NBN), the Fanconi pathway (FANCA, FANCL), and mismatch repair (MSH2, MSH6) suggests robust DNA repair circuits, which melanoma may exploit to maintain basal genomic stability during metastasis [59,63]. Conversely, downregulation of POLH and PRIMPOL, which are essential for DNA damage tolerance, particularly against UV-induced lesions, predisposes to melanoma [64,65,66,67].

In this context, heightened mitochondrial translation activity, with proteins involved in cristae formation, pyruvate metabolism, the tricarboxylic acid (TCA) cycle, and oxidative phosphorylation (OXPHOS), is also associated with tumor proliferation. Notably, two mitochondrial apoptotic pathways involving the activation and translocation of the BH3-only proteins NOXA and PUMA show a strong association with shorter progression-free survival (PFS). The upregulation of transcripts in these signaling axes may reflect a compensatory cellular response to an anti-apoptotic tumor microenvironment, indicating a dysregulated apoptotic program in melanoma. Interestingly, this metabolic reprogramming appears to contribute to both local recurrence and the development of distant metastases [68].

### 4.3. Phenotypic Switching and Invasive Phenotype

Our analysis highlighted a series of biological processes apparently poorly involved with melanocytes and melanomas, including the neuron projection arborization pathway (GO:0140058) and the myelination pathway (GO:0042552), which are dysregulated in all clusters compared to healthy samples, with no relevant differences among the subtypes.

Clinically, the reactivation of neurodevelopmental and Schwann-like programs in melanoma underscores the tumor’s ability to exploit its neural crest origin for phenotypic plasticity, immune evasion, and therapy resistance. Therapeutically, targeting pathways that sustain this reprogramming, particularly the AXL/YAP, IL-6/gp130, and COX-2–PGE2 axes, represent a promising approach to reduce melanoma adaptability and improve long-term treatment outcomes.

Both processes (neuron projection arborization and myelination) mediate typical functions of the nervous system, and their dysregulation in melanoma may represent a signal of reactivation of neuroectodermal programs associated with tumor plasticity and adaptation. These programs are associated with a switch toward *MITF*^low^/AXL^high^ phenotypes, which promote invasion, dissemination, and therapeutic adaptation [69]. Neurocrestal identity factors, such as TBX3, enhance transcriptional plasticity, increasing tumor formation and invasiveness [70]. Similarly, FABP7, characteristic of glial/neural progenitors, is frequently expressed in melanoma, promoting proliferation and invasion via PKC–MAPK/ERK signaling and integrating lipid metabolism with cell state [71].

Genes such as *SEMA6A* and *SEMA5A* orchestrate cytoskeletal remodeling and invasion. *SEMA6A* enables evasion of a dual BRAF/MEK blockade through a YAP-dependent pathway, while *SEMA5A* promotes progression, migration, and vasculogenic mimicry [72,73]. The Schwann-like signature likely reflects the crestal origin of melanocytes. Interestingly, previous studies [74,75] have reported that Schwann cells in the peritumoral context can be reprogrammed to an immunosuppressive, repair-like state, characterized by increased 12/15-LOX/COX-2 and PGE2/lipoxins, which suppress T-cell responses and facilitate tumor growth. While our data are consistent with the reactivation of such Schwann-like programs, the possibility of direct tumor–nerve crosstalk remains speculative and would require specific functional validation [76]. Finally, myelination programs seem to also be reactivated in some melanoma cells. Gp130 receptor signaling via IL-6/sIL-6R induces myelin-associated genes, including Po and MBP, driving transdifferentiation toward a myelinating glial phenotype.

This data provides insight into the plasticity of melanoma cells and the mechanisms by which neurodevelopmental programs support tumor adaptation and invasiveness [77], which appears to be heightened in the third subtype we highlighted. Monitoring such processes/genes could aid in monitoring tumor progression and potentially predict its invasiveness grade.

#### Hub Genes in Melanoma Proliferation/Invasive Pathways

To elucidate how hub genes regulate the balance between proliferative and invasive states in melanoma, we integrated our network analysis with both literature and transcriptomic evidence. Central to this axis are *MITF* and AXL, which act as antagonistic regulators of melanoma cell fate and therapy response. Within this framework, *MITF* functions as a lineage survival oncogene under MAPK and PI3K/AKT regulation [78]. Its suppression by Notch and the transcription factor *BRN2* promotes the transition to an invasive phenotype, whereas restoring *MITF* expression may resensitize tumors to MAPK inhibition.

Contrary to classical models of phenotype switching, our data reveal a concurrent upregulation of *MITF* and *BRN2*, while *AXL* expression remains unchanged.

Notably, *AXL*, *APAF1*, and *HIF1A*, which have been implicated in invasive behavior, apoptotic resistance, and hypoxia-driven plasticity, were not significantly dysregulated in any of the clusters. This indicates that the samples do not exhibit a full MITF-low/AXL-high or hypoxia-associated phenotype, but rather maintain a lineage-driven, proliferative transcriptional state, despite elevated *BRN2* expression.

Beyond these mechanisms, recent studies have identified the *MITF*/*APAF-1* axis as a determinant of MAPK inhibitor resistance, with *MITF* repressing APAF-1 and impairing apoptosome function [79]. Drug-repositioning approaches with quinacrine and methylbenzethonium were shown to inhibit *MITF* epigenetically, restore *APAF-1* activity, and resensitize resistant melanomas to MAPKi [80]. This highlights the dual role of MITF, which not only controls lineage identity and phenotypic plasticity but also modulates apoptotic escape. Regarding the elevated *BRN2* expression detected, the literature data support the possibility that *MITF* and *BRN2* can be co-expressed, particularly under certain microenvironmental or epigenetic conditions (e.g., adherent growth in vitro), without necessarily inducing a switch to an invasive program [81]. Nevertheless, it should be noted that this apparent co-expression may be the result of bulk RNA analysis, since it was observed that *BRN2*- and *MITF*-expressing tumor cells can coexist in the same tumor sample [82].

Collectively, these findings place *MITF* at the intersection of proliferation, invasion, and drug resistance, as summarized in Table 3.

### 4.4. Immune and Inflammatory Processes

Our analysis revealed that several immune- and inflammation-related pathways, including cellular response to cytokines (GO:0071345), inflammatory response (GO:0006954), cellular response to type II interferon (GO:0071346), T-helper cell lineage commitment (GO:0002295), and regulation of T-cell proliferation (GO:0042129) were found to be dysregulated in all clusters compared to control samples. This dysregulation appeared particularly evident for Cluster B, in which, other than the above cited BPs, the positive regulation of cytokine production (GO:0001819), B cell receptor signaling (GO:0050853), and T-cell activation and proliferation (GO:0042110; GO:0042129; GO:0050870) were also significantly dysregulated. The WGCNA revealed that these immune- and inflammation-related BPs were mainly associated with the White module. This module was negatively correlated with Cluster B and positively correlated with Cluster C.

Clinically, these results confirm that cutaneous melanoma subtypes maintain robust immune activity, consistent with their “hot” tumor phenotype and strong responsiveness to immunotherapy. However, variations in immune module regulation, particularly in Cluster B, indicate subtle but potentially meaningful differences in the balance between immune activation and suppression.

Therapeutically, this underscores the value of checkpoint inhibitors as a foundational strategy for all clusters, while suggesting that combination approaches, incorporating cytokine modulation, B cell pathway inhibition, or targeted therapies, may further enhance efficacy, particularly in tumors with partial immune dysfunction.

The sheer number of dysregulated processes and subprocesses demonstrates how immunity in its entirety (from innate to adaptative) and inflammatory processes play an important role in all melanoma subtypes, with a dysregulation in such functions within the tumor cells themselves.

The immunological phenotype of melanoma is closely linked to its molecular subtype. According to the Cancer Genome Atlas (TCGA) classification, cutaneous melanoma comprises four main subtypes: *BRAF*-mutant (~50%), *NRAS*-mutant (~20%), *NF1*-mutant (~10–15%) and triple negative/wild-type (~10–15%) [7].

*BRAF*-mutant melanomas tend to be immunologically “hot”, especially when associated with high tumor mutational burden (TMB). These tumors are generally sensitive to both targeted therapy and immunotherapy, particularly PD-1 inhibitors. Similarly, NRAS-mutant melanomas are also often associated with an inflammatory phenotype and are typically considered “hot” tumors. These tumors often present high levels of lymphocyte infiltration and respond well to immune checkpoint inhibitors, particularly anti-PD-1 agents. *NF1*-mutated melanomas also tend to be immunologically “hot”. In contrast, triple-negative or wild-type melanomas have a more heterogeneous immunological profile, although they are often classified as “cold” tumors, particularly in rare subtypes such as mucosal, acral lentiginous, and uveal melanomas [14].

**Figure 7:**
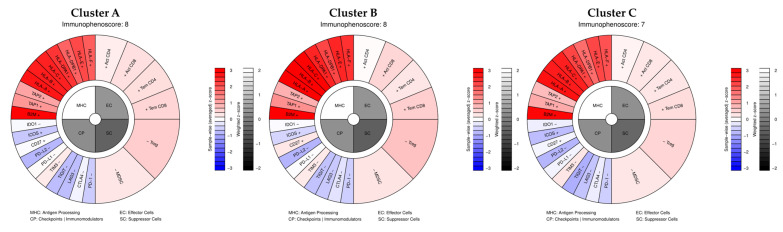
Averaged IPS scores of the three clusters and details of genes used for IPS calculation.

Our analysis shows that the average immunophenotypic score did not change much, with an IPS of 8 for clusters A and B and 7 for Cluster C (Figure 7), classifying the three clusters as “hot” and therefore more responsive to immunotherapy. Our data show that the transcriptional patterns defining the three clusters do not influence their immune profile. This is consistent with what is known about cutaneous melanomas, which, as seen, are generally hot tumors, compared to mucosal, acral lentiginous, and uveal melanomas, which are classified as cold.

The tumor microenvironment (TME) is shaped by complex interactions between tumor, immune, and stromal cells. Melanoma-derived exosomes activate tumor-associated fibroblasts (TAFs), inducing IL-6 and IL-8 secretion, reinforcing a pro-inflammatory phenotype that supports tumor progression [85,86]. Interferons mediate distinct responses: IFN-γ promotes the maturation of cDC2 cells, enhancing NK cytotoxicity in leptomeningeal metastases [86], while melanoma resistance to IFN-α involves STAT5 overexpression, counteracting STAT1-mediated antiproliferative signaling [87]. Under the influence of these inflammatory cytokines released in the TME, several immune cells can be polarized toward a pro-tumor or an anti-tumor phenotype, driven by a broad range of pro-tumorigenic and pro-angiogenic cytokines/chemokines. On the one hand, these cells can kill cancer cells via ROS and neutrophil elastase, induce apoptosis through TRAIL, inhibit angiogenesis through VEGF-A165b, and enhance T-cell immune responses upon TGF-β inhibition. On the other hand, the cells can promote tumor growth by releasing growth factors, induce genetic instability, remodel the extracellular matrix (ECM), support angiogenesis and lymphangiogenesis, and suppress anti-tumor immunity through mechanisms like arginine depletion and expression of PD-L1 [88].

For example, it has been observed that B cells display dual roles in melanoma. Regulatory B cells (Bregs), regulated by c-Maf and IL4I1, contribute to immunosuppression and tumor progression [89,90], whereas overall B cell activity remains context-dependent. CD8^+^ T cells, previously considered “exhausted,” are clonally expanded and dynamically proliferative within tumors, driving antitumor responses [91].

Neutrophils are highly plastic, capable of both antitumor and pro-tumor functions, including ROS-mediated killing, ECM remodeling, angiogenesis, and immune suppression. Elevated neutrophil-to-lymphocyte ratios (NLRs), increased degranulation, and neutrophil-mediated immunity are associated with inflammation, angiogenesis, tumorigenesis, and poorer prognosis [88,92,93].

Finally, tumor-associated macrophages (TAMs) modulate tumor survival, proliferation, and metastasis. While M1 macrophages are anti-tumorigenic, M2-like TAMs support inflammation, immune evasion, angiogenesis, and therapy resistance via STAT3 signaling [94].

Notably, inflammatory responses not only impact tumor development but also modulate the efficacy of immunotherapies. The inflammatory response score, calculated from tumor samples obtained during treatment, has emerged as a strong predictor of response to immune checkpoint inhibitors (ICIs) in patients with metastatic melanoma. Moreover, higher inflammatory response scores correlate with increased expression of immune checkpoint-related genes, suggesting that patients with elevated scores may experience enhanced benefit from ICI therapies [95].

### 4.5. General Alteration of Basic Cellular Functions

Our analysis identified dysregulation in fundamental cellular processes, including translation (GO:0006412), translational elongation (GO:0006414), and translational termination (GO:0006415). Translation and translational elongation were both enriched in the Lightcoral module, which was upregulated in Cluster C and downregulated in Cluster B. Translational elongation was also dysregulated in the Dimgrey module, together with translational termination, showing upregulation in Cluster A and downregulation in Cluster B. Overall, the data indicates a similar trend for Cluster A and C, in opposition to the trend observed in Cluster B.

The observed dysregulation of translational control mechanisms across melanoma subtypes reflects a fundamental reprogramming of the protein synthesis machinery that sustains tumor proliferation, adaptability, and therapeutic resistance. Clusters A and C, in particular, exhibit heightened translational activity, consistent with a metabolically active and aggressive phenotype.

Therapeutically, these findings support the exploration of translation-targeted therapies, RNA metabolism modulators, and combination regimens with existing targeted or immune-based treatments to overcome resistance and improve clinical outcomes in melanoma patients.

Normal cellular function and developmental processes require temporal and spatial regulation of gene expression by the activity of transcription factors that bind to the regulatory elements of each gene. Alteration and deletion resulting in the loss of activity of transcription factors have been linked to various human disorders, including cancer. For example, constitutive Notch activation induces the transcriptional repressor Hey1, which suppresses TweakR expression, reducing immune cell recruitment and facilitating tumor escape from immune surveillance. Conversely, blocking Hey1 enhances TweakR–CCL2 signaling, promoting immune cell recruitment while still supporting melanoma growth and metastasis [96].

Alterations in 3′ UTRs, such as the NBCn1 (SLC4A7) SNP rs4973768, have been linked to increased cancer risk [97], suggesting a possible contribution to melanoma susceptibility.

Maintaining a balance between mRNA production and decay is essential for homeostasis. Alterations in mRNA catabolism and NMD can lead to increased expression of oncogenic proteins and inactivation of tumor suppressors, particularly under hypoxia or oxidative stress, supporting tumor adaptation [98].

Finally, mRNA translation reprogramming plays a key role in melanoma survival and drug resistance. Rapino et al. showed that U34 enzymes are crucial for the survival of melanoma cells, maintaining high levels of HIF1α through codon-dependent regulation of HIF1A mRNA translation. Replacing the codon of HIF1A abolished the melanoma cells’ dependence on U34 enzymes and induced drug resistance in BRAFV600E melanoma [99].

On the other side, Wurth et al. demonstrated that the RNA-binding protein (RBP) UNR controls many of its targets at the level of translation elongation/termination, acting as an oncogenic modulator of melanoma progression [100].

Collectively, these alterations promote protein synthesis that supports tumor growth, survival, and metastasis.

### 4.6. Lipid Metabolism: Association and Roles Within Melanoma Progression

Our analysis revealed that lipid metabolic processes are significantly dysregulated in melanoma in all clusters. In this context, key enriched pathways include linoleic acid metabolism (GO:0043651), long-chain fatty acid metabolism and transport (GO:0001676; GO:0015909), and unsaturated fatty acid metabolism (GO:0033559), involving genes such as *FADS1/2, ELOVL3, PNPLA3, SLC27A2*, and *ALOX15B*.

Lipid metabolism, encompassing fatty acid synthesis/oxidation, cholesterol handling, and lipid-mediated signaling, is emerging as a critical regulator of cancer biology, including melanoma [101]. A key feature of cancer metabolic plasticity is the adaptive desaturation of fatty acids. Desaturases such as stearoyl-CoA desaturase (SCD) and fatty acid desaturase-2 (*FADS2*) are frequently dysregulated across tumor types. *FADS2* activation promotes epithelial–mesenchymal transition (EMT) and tumor progression in both patient samples and cell-line models, sustaining EMT plasticity through *FADS2*-mediated desaturation [102]. For example, in hepatocellular carcinoma, accumulation of 20:3n-9 reflects reduced Δ6-desaturase activity and increased arachidonic acid demand, consistent with metabolic reprogramming that may influence prognosis. Shifts toward monounsaturated fatty acids (MUFAs) have been linked to therapy resistance, with SCD-insensitive cells showing *FADS2* upregulation and increased sapienate (16:1n-10) synthesis, preserving membrane fluidity and lipid raft signaling, thereby promoting survival, invasion, and treatment tolerance [103,104]. Genomic and transcriptional alterations in this axis, including amplifications at 11q12-13 (housing *FADS1–FADS3*) and *FADS2*-derived circular RNAs, are associated with tumor aggressiveness and poor prognosis [105].

At the level of lipid metabolism, WGCNA also revealed changes in ELOVL3 expression behavior. Yu Zhang and colleagues have demonstrated that high ELOVL3 expression is associated with reduced overall survival (OS) and disease-free survival (DFS) in HCC, and this effect may be comparable in melanoma [106].

Unlike glycolysis or glutamine metabolism, lipid pathways influence proliferation, membrane biogenesis, oxidative stress buffering, and immune modulation [107,108]. Lipids also function as signaling molecules activating PI3K–AKT and MAPK pathways, potentially contributing to immune evasion. These findings underscore the central role of lipid metabolism in melanoma heterogeneity and suggest that therapeutic targeting of fatty acid synthase, CPT1A-mediated oxidation, or cholesterol pathways (e.g., statins) could be effective strategies.

### 4.7. Limits

The study suffers some limitations that should be taken into consideration for the interpretation of the results. To reduce computational load in this analysis we reduced the dimensionality of the expression matrix. Although this is a common and accepted approach, this step inevitably decreased the original expression data information. Thus, some expression patterns may have been lost. In future investigations we will try to increase the dimensionality to catch more variability. The data obtained was performed on a collection of different datasets (as E-MTAB-6697 is a collection of four databases) that may help generalize the results obtained from the analyses; nevertheless, further confirmation should be performed on different, independent samples to confirm our results. Our data was based on publicly available datasets that have little to no clinical information data available. As such, we cannot exclude the effect of possible confounders (including but not limited to comorbidities, previous therapies, or other concomitant treatments) in our analyses. This also means that clustering was strictly genetic-based, which, while aligned with our study focus, minimizes the contribution of environmental factors that may only indirectly influence expression patterns. Another limitation concerns the interpretation of ORA results for certain clusters. Cluster A showed no significant enrichment for any biological process (BPs), while Cluster C was enriched for only a single BP. Several factors may account for this observation. First, the ORA relies on currently available annotations, meaning that biological processes not yet characterized cannot be detected. Second, the dysregulated elements within these clusters may not converge on a single specific biological process but may instead act through broader regulatory mechanisms.

## 5. Conclusions

In conclusion, our analysis delineates the heterogeneity of melanoma at the transcriptional level, identifying three distinct molecular subtypes characterized by specific dysregulated processes. While all clusters shared dysregulation of pathways involved in epidermal differentiation, immune response, and lipid metabolism compared to healthy controls, they diverged in the extension of the dysregulation for proliferation, phenotypic plasticity, metabolic adaptation, and apoptotic regulation processes. In particular, Cluster C exhibited strong upregulation of genes related to cell cycle progression, and mitochondrial metabolism, pointing to a highly proliferative and plastic phenotype with invasive potential. Cluster A displayed activation of modules enriched in DNA replication, mitosis, and repair, as well as mitochondrial translation and metabolism, suggesting a proliferative yet genomically stable phenotype able to tolerate replication stress and dampen apoptotic signaling. Cluster B displayed relative downregulation of proliferative and translational programs and a complex rewiring of immune and inflammatory pathways compared to the other clusters. Despite these transcriptional differences, immunophenotype scores across clusters remain comparably high (IPS ≈ 8 for Clusters A and B, IPS ≈ 7 for Cluster C), indicating that most cases in our cohort retain an overall “hot” immune profile and therefore may remain amenable to immune-based therapies.

Across all subtypes, lipid metabolism is consistently dysregulated. This highlights lipid metabolism as a critical contributor to melanoma progression, phenotypic plasticity, and treatment tolerance, and suggests that targeting fatty acid synthesis, oxidation, or cholesterol handling may offer cluster-specific therapeutic opportunities. Overall, our study provides a cluster-resolved framework linking gene expression patterns, metabolic reprogramming, and functional consequences in melanoma, emphasizing the potential of lipid-focused interventions to improve personalized treatment strategies, while recognizing that these hypotheses require validation in independent cohorts and functional experiments.

## Figures and Tables

**Table 1:** Results from DEA. In the table are reported the number of samples labeled for each cluster and the number of controls used for comparison. The numbers of upregulated and downregulated DEGs are also reported.

Tumor Samples		Control Samples	Upregulated DEGs	Downregulated DEGs	Total DEGs
	N°	N°			
Cluster A	51	20	1606	1683	3289
Cluster B	52	20	860	1397	2257
Cluster C	59	20	2035	2134	4169

**Table 2:** Top five GO: BP associated with the clusters.

Term	Overlap	*p*-Value	Adjusted *p*-Value	Odds	Combined Score
				Ratio	
Cluster A-specific					
-	-	-	-	-	-
Cluster B-specific					
Positive regulation of T-cell activation (GO:0050870)	13/111	1.53 × 10^−8^	2.02 × 10^−5^	8.87 × 10^00^	1.60 × 10^2^
Lymphocyte differentiation (GO:0030098)	9/76	2.13 × 10^−8^	2.02 × 10^−5^	1.13 × 10^1^	1.99 × 10^2^
Antigen receptor-mediated signaling pathway (GO:0050851)	12/114	1.82 × 10^−7^	1.16 × 10^−4^	7.84 × 10^00^	1.22 × 10^2^
B cell-mediated immunity (GO:0019724)	6/20	3.95 × 10^−7^	1.88 × 10^−4^	2.81 × 10^1^	4.15 × 10^2^
Regulation of Interleukin-12 Production (GO:0032655)	8/50	8.42 × 10^−7^	2.89 × 10^−4^	1.26 × 10^1^	1.76 × 10^2^
Cluster C-specific					
Regulation of intracellular signal transduction (GO:1902531)	38/302	5× 10^−6^	0.0169708	2.40123	29.29741
Common					
Epidermis development (GO:0008544)	38/86	1.07 × 10^−19^	4.09 × 10^−16^	9.32 × 10^00^	4.07 × 10^2^
Intermediate filament organization (GO:0045109)	32/72	6.77 × 10^−17^	1.29 × 10^−13^	9.39 × 10^00^	3.49 × 10^2^
Supramolecular fiber organization (GO:0097435)	66/339	9.05 × 10^−12^	1.15 × 10^−8^	2.86 × 10^00^	7.28 × 10^1^
Long-chain fatty acid metabolic process (GO:0001676)	26/85	1.16 × 10^−9^	1.02 × 10^−6^	5.15 × 10^00^	1.06 × 10^2^
Very long-chain fatty acid metabolic process (GO:0000038)	15/30	1.57 × 10^−9^	1.02 × 10^−6^	1.16 × 10^1^	2.36 × 10^2^

**Table 3:** Summarizes the integration of hub genes into melanoma-relevant pathways, combining literature-based mechanisms with our transcriptomic evidence. *MITF* emerged as consistently higher than *AXL* across clusters, indicating a predominantly proliferative state, while BRN2 was weakly expressed and *HIF1A* highly expressed. The inclusion of the *MITF/APAF-1* axis highlights how apoptosis regulation contributes to MAPK inhibitor resistance and identifies potential therapeutic vulnerabilities.

Hub Gene/Axis	Linked Pathway(s)	Mechanistic Role (Literature)	Evidence from Our Dataset	Functional Hypothesis	References
*MITF*	MAPK, PI3K/AKT, EMT	Lineage survival oncogene; rheostat model; suppressed by Notch/BRN2 → invasive switch	Higher than AXL across clusters (MITF-high/AXL-low); Cluster B with reduced MITF suggests transitional state	Restoring MITF activity could resensitize tumors to MAPKi	[78]
*AXL*	EMT, PI3K/AKT, drug resistance	Marker of MITF-low phenotype; promotes invasion and MAPKi resistance	Lower than MITF in all clusters; no AXL-driven subgroup detected	AXL inhibition may counteract EMT-like resistance	[83]
*BRN2 (POU3F2)*	MAPK, EMT, Notch cross-talk	Antagonist of MITF; cooperates in AXL regulation; drives invasion	Weakly expressed; not a cluster driver	Targeting BRN2 may restore MITF expression and reduce invasion	[84]
*NGFR (p75NTR)*	EMT, stemness, immune evasion	Marker of neural crest-like MITF-low state; supports plasticity and immune escape	Not strongly represented in our dataset	Targeting NGFR-positive subpopulations could limit relapse and immune evasion	[83]
*MITF/APAF-1* axis	Apoptosis, MAPK inhibitor resistance	MITF represses APAF-1 → impaired apoptosome and resistance	Not directly clustered, but consistent with MITF dominance	Pharmacologic inhibition of MITF or APAF-1 reactivation (quinacrine, MBZ) may restore apoptosis and sensitize tumors	[79,80]

## Data Availability

Data is contained within the article or Supplementary Materials.

## Supplementary Materials

The following supporting information can be downloaded at https://www.mdpi.com/article/10.3390/genes16121428/s1, Figure S1: Clustering of module eigen genes, Tables S1 and S2: Comprehensive list of all enriched GO terms, along with their associated DEGs.

## Abbreviations

The following abbreviations are used in this manuscript:

CM	Cutaneous Melanoma
RR	Relative Risk
GEO	Gene Expression Omnibus
UMAP	Uniform Manifold Approximation and Projection
PCA	Principal Component Analysis
t-SNE	t-Distributed Stochastic Neighbor Embedding
CDF	Cumulative Distribution Function
DEA	Differential Expression Analysis
DEGs	Differentially Expressed Genes
FDR	False Discovery Rate
GO	Gene Ontology
WGCNA	Weighted-Gene Co-Expression Network Analysis
RP	Rank Product
ORA	Over-Representation Analysis
BP	Biological Process
GO:BP	Gene Ontology: Biological Processes
TCA	Tricarboxylic Acid
OXPHOS	Oxidative Phosphorylation
PFS	Progression-Free Survival
TME	Tumor Microenvironment
TAFs	Tumor-Associated Fibroblasts
ECM	Extracellular Matrix
Bregs	Regulatory B Cells
NLR	Neutrophil-To-Lymphocyte Ratios
TAMs	Tumor-Associated Macrophages
ICIs	Immune Checkpoint Inhibitors
NMD	Nonsense-Mediated Decay
RBP	RNA-Binding Protein
ALA	Alpha-Linolenic Acid
SCD	Stearoyl-Coa Desaturase
FADS2	Fatty Acid Desaturase-2
EMT	Epithelial–Mesenchymal Transition
MUFAs	Monounsaturated Fatty Acids
FADS1	Fatty Acid Desaturase 1
FADS3	Fatty Acid Desaturase 3
OS	Overall Survival
DFS	Disease-Free Survival
FAO	Fatty Acid Oxidation
