# Supplementary figures and images for: Genetic Insight into Expression-Defined Melanoma Subtypes and Network Mechanisms: An in Silico Study
Source: Genes (Basel). 2025 Nov 30;16(12):1428. doi: 10.3390/genes16121428 (PMC12732661; doi:10.3390/genes16121428)

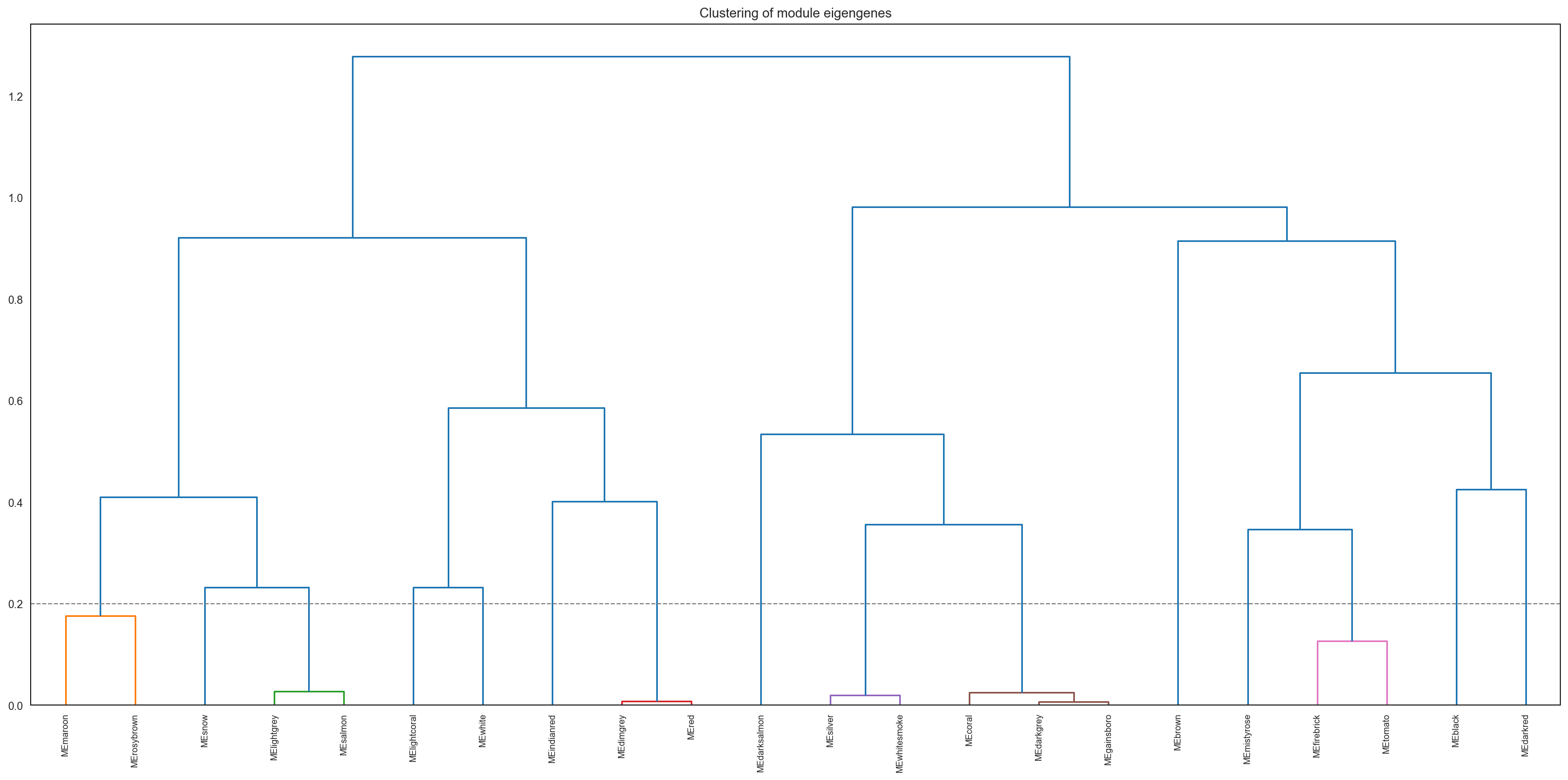

Supplement: Supplementary file 1 [file genes-16-01428-s001.zip › Supplementary Figure S1.png]
